# Characterization of osimertinib (AZD9291)-resistant non-small cell lung cancer NCI-H1975/OSIR cell line

**DOI:** 10.18632/oncotarget.13150

**Published:** 2016-11-07

**Authors:** Zheng-Hai Tang, Xiao-Ming Jiang, Xia Guo, Chi Man Vivienne Fong, Xiuping Chen, Jin-Jian Lu

**Affiliations:** ^1^ State Key Laboratory of Quality Research in Chinese Medicine, Institute of Chinese Medical Sciences, University of Macau, Macao, China

**Keywords:** osimertinib, AZD9291, EGFR, navitoclax, NSCLC

## Abstract

Osimertinib (OSI, also known as AZD9291) is the newest FDA-approved epidermal growth factor receptor (EGFR) tyrosine kinase inhibitor for non-small cell lung cancer (NSCLC) patients with EGFR T790M mutation. However, resistance to OSI is likely to progress and the study of potential OSI-resistant mechanisms in advanced is necessary. Here, the OSI-resistant NCI-H1975/OSIR cells were established. After cells developed resistance to OSI, cell proliferation was decreased while cell migration and invasion were increased. The NCI-H1975/OSIR cells exhibited more resistance to gefitinib, erlotinib, afatinib, rociletinib, doxorubicin, and fluorouracil, meanwhile showing higher sensitivity to paclitaxel, when compared with NCI-H1975 cells. In addition, the NCI-H1975/OSIR cells did not display multidrug resistance phenotype. The activation and expression of EGFR were decreased after cells exhibited resistance. Compared with NCI-H1975 cells, the activation of ERK and AKT in NCI-H1975/OSIR cells could not be significantly inhibited by OSI treatment. Navitoclax (ABT-263)-induced cell viability inhibition and apoptosis were more significant in NCI-H1975/OSIR cells than that in NCI-H1975 cells. Moreover, these effects of navitoclax in NCI-H1975/OSIR cells could be reversed by pretreatment of Z-VAD-FMK. Collectively, loss of EGFR could pose as one of the OSI-resistant mechanisms and navitoclax might be the candidate drug for OSI-resistant NSCLC patients.

## INTRODUCTION

Non-small cell lung cancer (NSCLC), which accounts for about 85% of lung cancer, is one of the most common cancers and a leading cause of cancer-related death in the world [1, 2]. The current survival rate of NSCLC still remains low despite significant improvements in molecular diagnosis and therapy strategies [1, 3]. The epidermal growth factor receptor (EGFR), anaplastic lymphoma kinase (ALK), and c-ros oncogene 1 (ROS1) *etc.*, are current molecular biomarkers that directly impact clinical therapy strategies for NSCLC [3]. Among these biomarkers, EGFR is one of the most extensively studied molecular subsets and about 10-40% of NSCLC patients harbor activating mutations of EGFR (mainly includes a deletion in exon 19 and/or a point mutation in exon 21) [4, 5]. Patients with these mutations frequently experience enhanced kinase activity of EGFR as well as good initial responses to the established EGFR tyrosine kinase inhibitors (TKIs), such as gefitinib, erlotinib, and afatinib *etc.* [6, 7]. Unfortunately, most patients will eventually experience resistance to these EGFR TKIs, with disease progression approximately 12 months after treatment [7, 8]. Multiple molecular mechanisms of resistance to EGFR TKIs have been identified in clinical NSCLC patients, such as second mutation of EGFR, amplification of MET, small cell histologic transformation, and epithelial mesenchymal transition [9-11]. Among these resistant mechanisms, second mutation of EGFR (T790M mutation, the gate keeper position of the kinase domain of EGFR) is best characterized and most commonly occurring, observed in 60% of EGFR-mutant NSCLC patients with acquired resistance to gefitinib and erlotinib [9]. In order to specifically target T790M mutation and sensitive mutation of EGFR, numerous of third generations of EGFR TKIs are being developed, such as osimertinib (OSI), rociletinib (also known as CO-1686), and WZ4002 [12, 13].

OSI is an oral and irreversible EGFR TKI with high selectivity against patients harboring EGFR sensitive mutation and T790M resistant mutation [12]. Compared with previous EGFR TKIs, OSI exhibited remarkably higher activity against EGFR with T790M versus against wild-type EGFR [12]. Clinical studies indicated that OSI (20 to 240 mg/day) was highly effective in NSCLC patients harboring EGFR T790M mutation who experienced disease progression during prior therapies with gefitinib or erlotinib. The median progression-free survival of patients with EGFR T790M-positive mutation was 9.6 months, meanwhile only 2.8 months in EGFR T790M-negative patients, and no dose-limiting toxicities were observed [13]. Due to the effectiveness of OSI in EGFR T790M mutation NSCLC patients, OSI is currently the only FDA-approved third generation of EGFR TKI for NSCLC patients with EGFR T790M positive mutation. So far, various clinical trials of OSI are being conducted, such as the therapeutic effects of OSI versus gefitinib or erlotinib in EGFR-TKI sensitive mutation of naive NSCLC patients [14] and the comparison of OSI with doublet chemotherapy (carboplatin and pemetrexed) as second-line therapy strategy for patients with advanced EGFR T790M NSCLC patients [15]. However, past history with FDA-approved EGFR TKIs suggests that there is likelihood for resistance to OSI to develop which can potentially restrict its therapy effects. Therefore, identifying possible resistant mechanisms of OSI in advance is important to provide a basis for the development of new therapeutic strategies for OSI-resistant patients.

In the present study, OSI-resistant cells (NCI-H1975/OSIR) were developed and the biological properties and potential resistant mechanisms were characterized to shed light on possible therapeutic strategy against OSI-resistance.

## RESULTS

### Establishment of NCI-H1975 cells resistant to OSI

**Figure 1:**
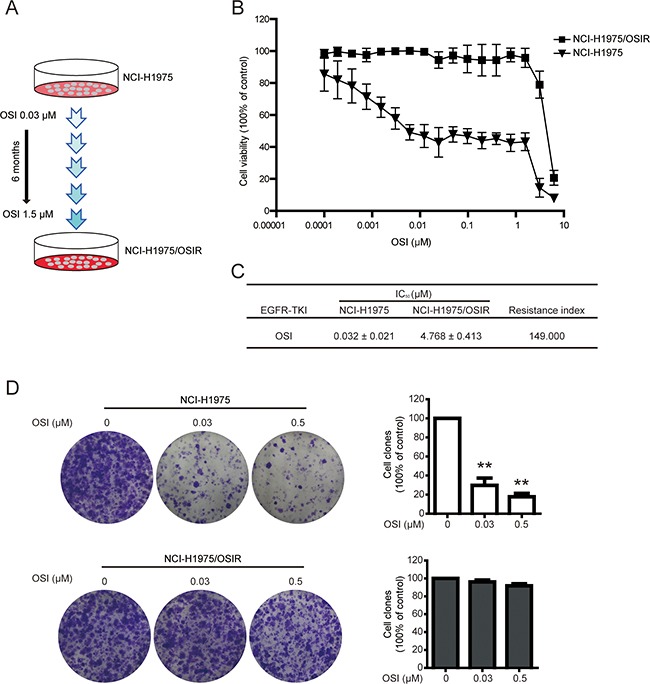
Establishment of NCI-H1975 cells resistant to OSI **A.** Schematic of establishing OSI-resistant NCI-H1975 cells. **B.** Cells were incubated with various concentrations of OSI for 72 h. The anti-proliferative effects of OSI in NCI-H1975 and NCI-H1975/OSIR cells were evaluated by MTT assay. *P<0.05 and **P<0.01, compared with the 0 μM OSI treatment. **C.** The IC_50_ values of OSI in NCI-H1975 and NCI-H1975/OSIR cells. **D.** Cells were exposed to OSI for 72 h and incubated with drug-free medium for 7 days. Then, cells were fixed with 4% PFA and stained with crystal violet. The cell colonies were photographed and representative images were exhibited. For quantitative assay, clones were dissolved in acetate acid after crystal violet staining and absorbance was recorded. *P<0.05 and **P<0.01.

NCI-H1975/OSIR cells were established from NCI-H1975 cells through dosage-escalation of OSI from 0.03 μM to 1.5 μM for about 6 months (Figure 1A). The cell viabilities of NCI-H1975 and NCI-H1975/OSIR cells following OSI treatment were studied by 3-(4,5-dimethylthiazol-2-yl)-2, 5-Diphenyltetrazolium bromide (MTT) assay. The cell viability of NCI-H1975/OSIR cells did not decrease as significantly as that of NCI-H1975 cells after exposure to OSI for 72h (Figure 1B). The IC_50_ values of OSI for NCI-H1975 and NCI-H1975/OSIR cells were 0.03 μM and 4.77 μM, respectively (Figure 1C). To further confirm the resistant property of NCI-H1975/OSIR cells to OSI, the colony formation abilities of NCI-H1975 and NCI-H1975/OSIR cells after treatment with OSI were detected. Treatment of NCI-H1975 cells with 0.03 μM and 0.5 μM OSI decreased the cell colony formation. However, the colony formation of NCI-H1975/OSIR cells was not decreased after treatment with OSI, even at the concentration of 0.5 μM OSI (Figure 1D).

### Characterization of the proliferation, migration, and invasion abilities of NCI-H1975 and NCI-H1975/OSIR cells

**Figure 2:**
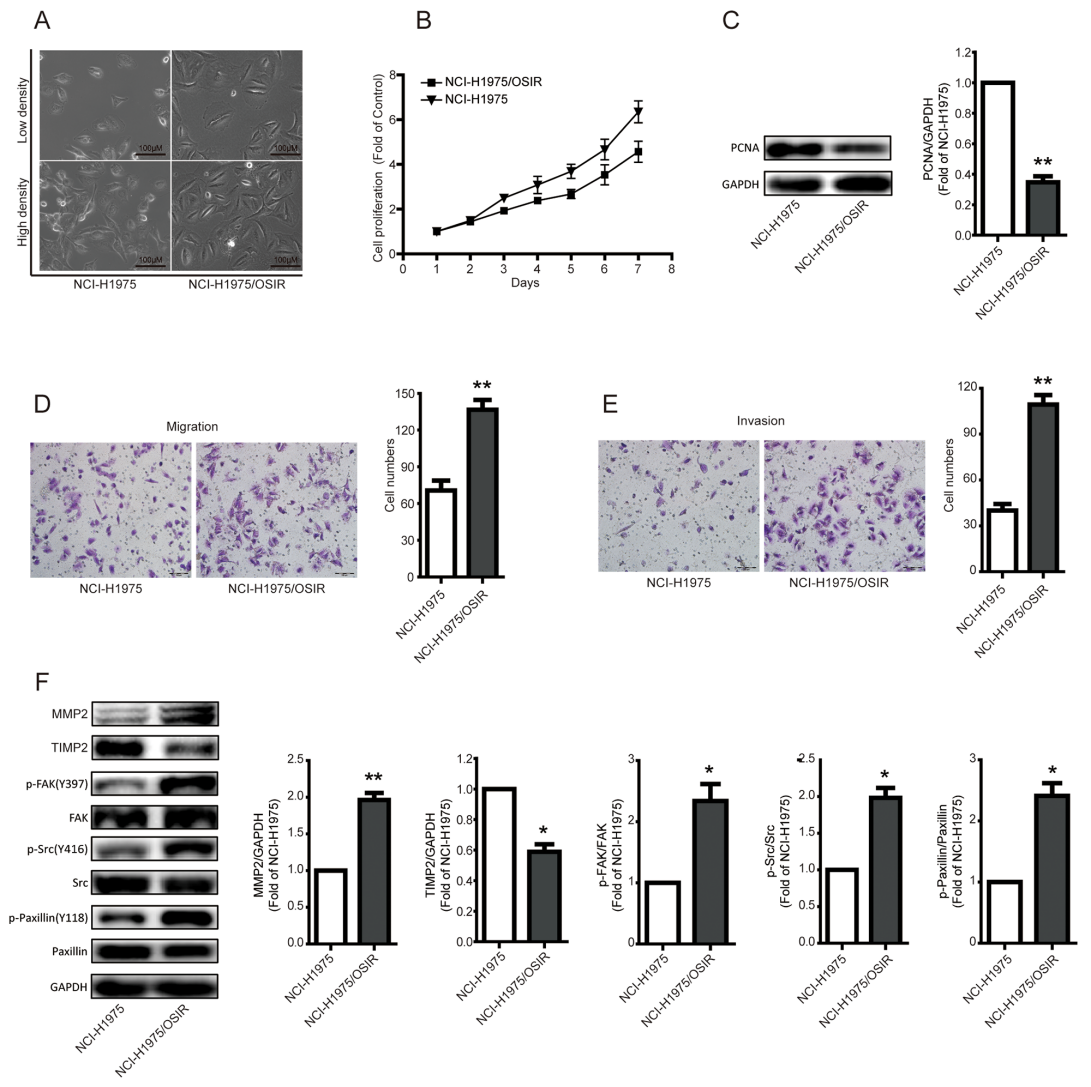
Characterization of the proliferation, migration, and invasion abilities of NCI-H1975 and NCI-H1975/OSIR cells **A.** After cells adhered to 6-well plate for 24h, the cell morphology was imaged and typical photos were presented. **B.** After NCI-H1975 and NCI-H1975/OSIR cells were plated in 96-well plate at same concentration, the cell proliferation was detected by MTT assay as indicated days. **C.** The protein expression of PCNA was determined by western blot assay. *P<0.05 and **P<0.01. **D, E.** the migration and invasion of NCI-H1975 and NCI-H1975/OSIR cells were determined by transwell assay. The migration and invasion ability of cells was determined by counting cell numbers that migrated or invaded the underside of the porous polycarbonate membrane by crystal violet staining. Typical images were photographed and presented. The quantification result was obtained from three independent experiments. *P<0.05 and **P<0.01. **F.** The expressions of indicated proteins were determined by western blot assay. *P<0.05 and **P<0.01.

After long-term exposure to OSI, great changes in cell morphology, proliferation, migration, and invasion were observed in NCI-H1975 cells. As shown in Figure 2A, NCI-H1975/OSIR cells have a bigger cell size and more fibroblast-like cell shape, compared with NCI-H1975 cells. The cell proliferation ability of NCI-H1975 and NCI-H975/OSIR cells from day 1 to day 7 without any treatment was studied by MTT assay. NCI-H1975/OSIR cells grew more slowly than NCI-H1975 cells, with proliferation rates of 149.41%, 249.36%, 308.20%, 369.06%, 466.46%, and 634.87% from Day1 to Day 7 respectively for NCI-H1975 cells and 143.36%, 193.02%, 238.44%, 267.99%, 353.82%, and 456.70% from Day 1 to Day 7 respectively for NCI-H1975/OSIR cells (Figure 2B). The protein expression of PCNA, which is essential for cell replication [16], decreased after NCI-H1975 cells obtained resistance to OSI (Figure 2C). In addition, the cell migration (without matrigel) and invasion (with matrigel) abilities of each cell line, which are crucial for cell metastasis [17], were evaluated using transwell assays. As shown in Figure 2D and E, more NCI-H1975/OSIR cells than NCI-H1975 cells passed through the upper membrane of the transwell inserts, regardless of whether the inserts were pretreated with matrigel. Considering the activations of FAK, Src, and Paxillin are critical for cell metastasis [18], western blot analysis was preformed and results indicated that the activations of FAK, Src, and Paxillin were higher in NCI-H1975/OSIR cells, compared with NCI-H1975 cells (Figure 2F). Furthermore, increased expression of MMP2 and decreased expression of TIMP2 proteins have been strongly associated with cell metastasis [19]. As shown in Figure 2F, MMP2 expression was higher while the TIMP2 expression was lower in NCI-H1975/OSIR cells, compared with NCI-H1975 cells. The aforementioned results demonstrated that NCI-H1975/OSIR cells exhibited slower cell proliferation rate while higher cell migration and invasion abilities when compared with NCI-H1975 cells.

### Characterization of the sensitivity of NCI-H1975 and NCI-H1975/OSIR cells to EGFR TKIs and chemotherapeutics

**Figure 3:**
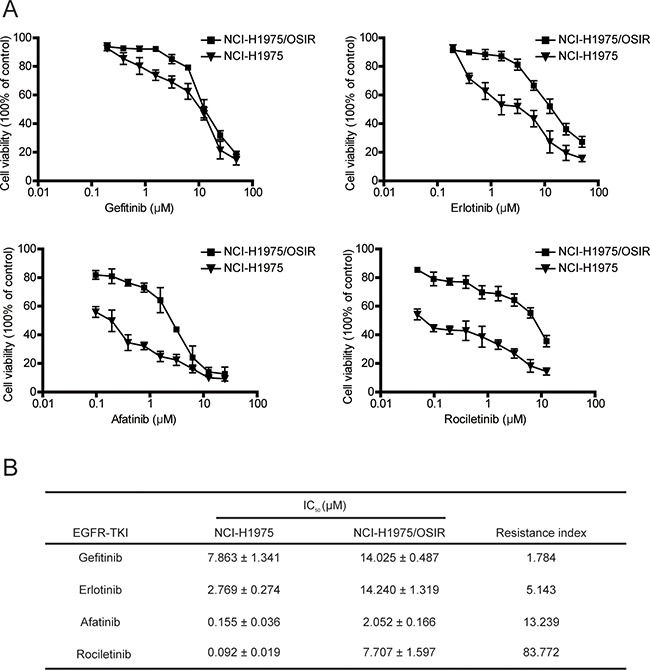
Characterization of the sensitivity of NCI-H1975 and NCI-H1975/OSIR cells to EGFR TKIs **A.** NCI-H1975 and NCI-H1975/OSIR cells were treated with indicated concentrations of EGFR TKIs for 72 h. Cell viability was studied using MTT assay. **B.** The IC_50_ values of EGFR TKIs in NCI-H1975 and NCI-H1975/OSIR cells.

**Figure 4:**
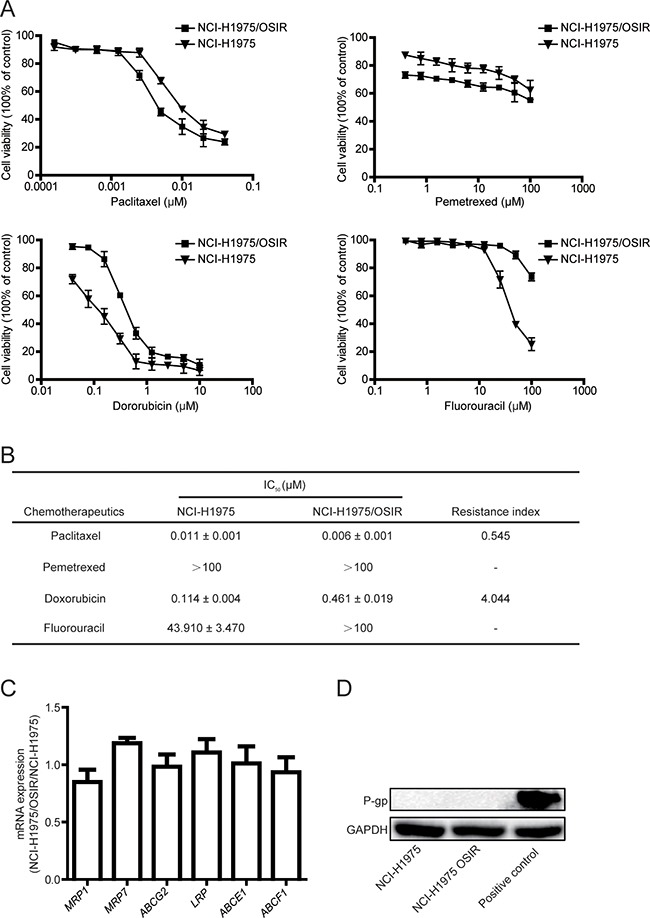
Characterization of the sensitivity of NCI-H1975 and NCI-H1975/OSIR cells to chemotherapeutics **A.** After cells were exposed to various concentrations of chemotherapeutics for 72 h, the cell viability was detected by MTT assay. **B.** The IC_50_ values of chemotherapeutics in NCI-H1975 and NCI-H1975/OSIR cells. “-” means “cannot statistics”. **C.** The mRNA levels of indicated genes in NCI-H1975 cells and NCI-H1975/OSIR cells were determined by RT-PCR. **D.** The cell proteins were extracted and the expressions of P-gp and GAPDH were studied by western blot.

Therapy strategies after emergence of EGFR TKIs resistance has been a controversial issue. The decision of whether other EGFR TKIs could be employed upon resistance remains unclear [20]. In the present study, the FDA-approved EGFR TKIs (the first generation EGFR TKIs gefitinib and erlotinib and the second generation EGFR TKI afatinib) were employed to study the anti-proliferation in NCI-H1975 and NCI-H1975/OSIR cells. NCI-H1975/OSIR cells were more tolerant to EGFR TKIs than NCI-H1975 cells, with resistance indexes of 1.784, 5.143, and 13.239 for gefitinib, erlotinib, and afatinib, respectively (Figure 3A and 3B). Furthermore, the third generation EGFR TKI rociletinib, which is currently under clinical trial [21], was also studied. The half maximal inhibitory concentration (IC_50_) of rociletinib in NCI-H1975/OSIR cells (7.707 μM) was higher than that in NCI-H1975 cells (0.092 μM) (Figure 3A and 3B). The chemotherapeutics are also drug options for treatment of the EGFR TKIs-resistant patients [22]. The anti-proliferation of chemotherapeutics in NCI-H1975 and NCI-H1975/OSIR cells were detected. As shown in Figure 4A and 4B, NCI-H1975/OSIR cells were more sensitive to paclitaxel than NCI-H1975 cells. Although cells resistance to OSI showed less resistant to pemetrexed, the anti-proliferation of pemetrexed in NCI-H1975/OSIR cells was still weak (Figure 4A and 4B). Compared with NCI-H1975 cells, NCI-H1975/OSIR cells were more resistant to doxorubicin (IC_50_, 0.114 μM in NCI-H1975 cells and 0.461 μM in NCI-H1975/OSIR cells) and fluorouracil (IC_50_, 43.910 μM in NCI-H1975 cells and more than 100 μM in NCI-H1975/OSIR cells) (Figure 4A and 4B). In addition, the mRNA expressions of multiple drug resistance (MDR) genes, such as *MRP1*, *MRP7*, *ABCG2*, *LRP*, *ABCE1*, and *ABCF1*, were detected by RT-PCR assay. Result indicated that the mRNA levels of MDR genes in NCI-H1975 and NCI-H1975/OSIR cells were similar (Figure 4C). Moreover, the protein expression of P-gp in both cell lines was unexpressed (Figure 4D).

### Characterization of EGFR and the downstream proteins in NCI-H1975 and NCI-H1975/OSIR cells

**Figure 5:**
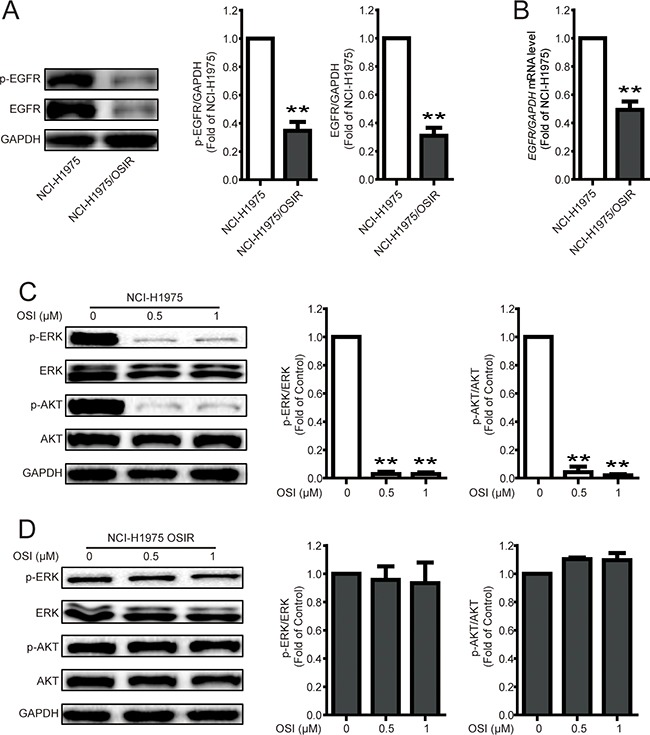
Characterization of EGFR and the downstream proteins in NCI-H1975 and NCI-H1975/OSIR cells **A.** After cells were plated for 24 h, the cell proteins were extracted and the expressions of p-EGFR, EGFR, and GAPDH were studied by western blot. *P<0.05 and **P<0.01. **B.** The mRNA levels of EGFR in NCI-H1975 and NCI-H1975/OSIR cells were determined by RT-PCR. *P<0.05 and **P<0.01. **C-D.** NCI-H1975 and NCI-H1975/OSIR cells were cultured with indicated concentrations of OSI for 24 h. Cell extracts were analyzed for indicated protein expression using western blot analysis. The blots of Figure 5C and 5D were obtained under the same exposure time. *P<0.05 and **P<0.01.

Although various mechanisms are responsible for EGFR TKIs resistance, studies related to the resistant mechanisms of the third generation EGFR TKI OSI are rare and unclear [12, 23]. Here, NCI-H1975/OSIR cells exhibited lower phosphorylated and total expressions of EGFR than that in NCI-H1975 cells (Figure 5A). Furthermore, the mRNA expression of EGFR in NCI-H1975/OSIR cells was only 0.49 fold of that in NCI-H1975 cells (Figure 5B). ERK and AKT are two major downstream proteins of the EGFR pathway and inhibition of the EGFR pathway can decrease the phosphorylation of ERK and AKT [24]. In this study, OSI significantly inhibited the phosphorylation of ERK and AKT in NCI-H1975 cells while not in NCI-H1975/OSIR cells (Figure 5C and 5D). It can be concluded that the down-regulation of EGFR expression might contribute to the resistance of OSI in NCI-H1975/OSIR cells.

### Characterization of sensitivity to navitoclax in NCI-H1975 and NCI-H1975/OSIR cells

**Figure 6:**
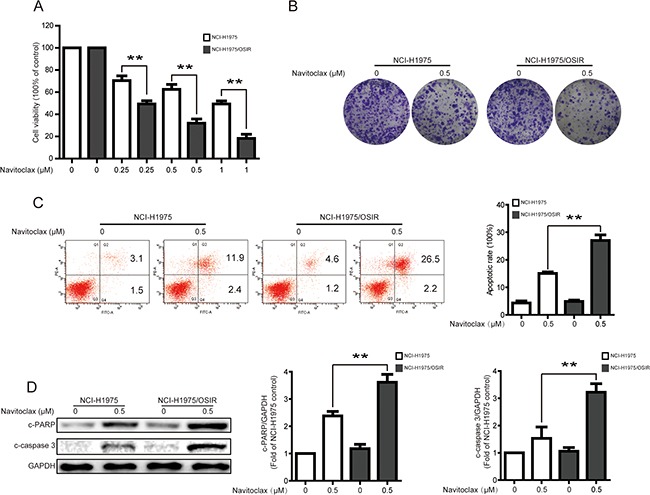
Characterization of sensitivity to navitoclax in NCI-H1975 and NCI-H1975/OSIR cells **A.** NCI-H1975 and NCI-H1975/OSIR cells were treated with various concentrations of navitoclax for 48 h. Cell viability was evaluated using MTT assay. *P<0.05 and **P<0.01. **B.** After cells were incubated with 0.5 μM navitoclax for 48 h, NCI-H1975 and NCI-H1975/OSIR cells were cultured with drug-free medium for 7 days. Cell colonies were stained with crystal violet and photographed. The representative images were exhibited. **C.** NCI-H1975 and NCI-H1975/OSIR cells were treated with 0.5 μM navitoclax for 48 h. the apoptotic cells were stained with annexin V/PI and analyzed by a flow cytometry according to the manufacturer's instructions. *P<0.05 and **P<0.01. **D.** After treatment with 0.5 μM navitoclax for 48 h, cells were analyzed to determine indicated changes of proteins by western blot analysis. *P<0.05 and **P<0.01.

The decreased expression of EGFR in cells have been reported to be sensitivity to the BH3 mimetic navitoclax [11]. Therefore, the cell viabilities of NCI-H1975 and NCI-H1975/OSIR cells following navitoclax treatment were studied. As shown in Figure 6A, cells were treated with various concentrations of navitoclax for 48 h. The cell viability for the NCI-H1975/OSIR cells were significantly lower than that for the NCI-H1975 cell line, with cell viabilities remaining at 70.48%, 62.57%, and 49.35% for NCI-H1975 cells and 49.35%, 31.94%, and 18.27% for NCI-H1975/OSIR cells after treatment with 0.25, 0.5, and 1 μM navitoclax, respectively. This difference in cell viability inhibition of navitoclax in NCI-H1975 and NCI-H1975/OSIR cells were further confirmed by colony formation analysis. Fewer colonies were observed in NCI-H1975/OSIR cells after treatment with navitoclax, when compared with NCI-H1975 cells (Figure 6B). In order to investigate the potential mechanisms for the aforementioned phenomenon, the pro-apoptotic effect of navitoclax in the two cell lines was studied. Annexin V-FITC and propidium iodide (PI) dual labeling assay demonstrated that a higher percentage of apoptotic cell was induced by navitoclax treatment in NCI-H1975/OSIR cells than in NCI-H1975 cells (Figure 6C). In addition, the protein expressions of c-PARP and c-caspase 3, which are biomarkers of apoptosis, were studied via western blot. As shown in Figure 6D, the navitoclax-induced expressions of c-PARP and c-caspase 3 proteins were more remarkable in NCI-H1975/OSIR cells, compared with NCI-H1975 cells. These data indicated that NCI-H1975/OSIR cells were more sensitive to navitoclax, compared with NCI-H1975 cells.

### Navitoclax-induced cell viability inhibition and apoptosis can be reversed by Z-VAD-FMK in NCI-H1975/OSIR cells

**Figure 7:**
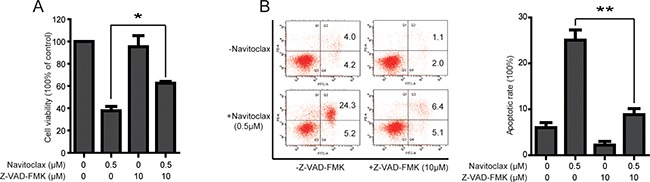
Navitoclax-induced cell viability inhibition and apoptosis can be reversed by pretreatment of Z-VAD-FMK in NCI-H1975/OSIR cells **A.** NCI-H975/OSIR cells were treated with navitoclax for 48 h with or without pretreatment of Z-VAD-FMK (10 μM, 1 h). Cell viability was then evaluated using MTT assay. *P<0.05 and **P<0.01. **B.** After pretreatment with 10 μM Z-VAD-FMK for 1 h, NCI-H1975/OSIR cells were incubated with 0.5 μM navitoclax for 48 h, the apoptotic cells were stained with annexin V/PI and determined by a flow cytometry according to the manufacturer's instructions. *P<0.05 and **P<0.01.

To further confirm that navitoclax-induced decrease in cell viability is caused by navitoclax-induced apoptosis through activation of caspase in NCI-H1975/OSIR cells. The cell viability and apoptosis were detected after pretreatment with the pan-caspase inhibitor benzyloxycarbonyl-Val-Ala-Asp (OMe) fluoromethylketone (Z-VAD-FMK). As shown in Figure 7A, the cell viability in navitoclax alone-treated group remained at 37.75% while the cell viability in navitoclax and Z-VAD-FMK combination-treated group was 62.50%. Annexin-FITC and PI staining indicated that navitoclax-increased cell apoptosis was reversed from 25.07% to 8.83% after pretreatment with 10 μM Z-VAD-FMK for 1 h in NCI-H1975/OSIR cells.

## DISCUSSION

NSCLC patients with EGFR positive mutations can benefit from EGFR TKIs such as gefitinib, erlotinib, and afatinib. However, most patients will experience resistance to these EGFR TKIs after approximately 12 months of treatment. So far, OSI is the only US FDA-approved third generation EGFR TKI for NSCLC patients with EGFR T790M mutation experiencing disease progression after treatment with EGFR TKIs. As resistance to OSI is likely to occur, the advance identification of possible resistant mechanisms of OSI and developing effective strategies for OSI-resistant patients are necessary. In this study, we established an OSI-resistant cell line, NCI-H1975/OSIR cells, by dosage-escalation. The biological properties of NCI-H1975/OSIR cells in terms of proliferation, migration, invasion, and sensitivity to other EGFR TKIs and chemotherapeutics were studied. Decrease of EGFR expression might be a resistant mechanism for OSI in NCI-H1975/OSIR cells and navitoclax could be a potential candidate drug for OSI-resistant NSCLC patients.

Cancer cells activate the growth-promoting signals to maintain sustained cell proliferation [25]. Here, the proliferation of NCI-H1975/OSIR cells was slower than that of the parent cells, indicating that certain proliferative signals, for example the EGFR pathway, in NCI-H1975/OSIR cells might be damaged. In addition, increased abilities of migration and invasion of the resistant cells indicated that they might possess a higher metastatic ability, which is one of the characteristics of cancer malignancy [25]. Therefore, cells might gain malignancy during acquisition of resistance to OSI. The migration and invasion abilities of NSCLC cells were also increased after cells acquired resistance to EGFR TKI gefitinib [26]. Therefore, increased metastatic potential of cancer cells should be considered during treatment with EGFR TKIs in NSCLC patents.

ALK expressed in about 4-6% in NSCLC patients, is also an important molecular biomarker for NSCLC patients [27, 28]. Recently, an *in vivo* study indicated that first generation of ALK inhibitor crizotinib could significantly overcome the resistance to second generation of ALK inhibitor alectinib [29]. Thus, the sensitivity of NCI-H1975/OSIR cells to first, second, and other third generation EGFR TKIs were evaluated. Unfortunately, NCI-H1975/OSIR cells showed resistance to all these EGFR TKIs, indicating that gefitinib, erlotinib, afatinib as well as rociletinib might not be effective for OSI-resistant patients. Comparing the treatment effects of OSI versus gefitinib or erlotinib in EGFR-TKI sensitive mutation of naive NSCLC patients was currently in clinical studying [14]. The overall survival of patients with EGFR sensitive mutation who were first treated with gefitinib or erlotinib, then with OSI after resistance occurred, was about 21 moths [7, 13]. However, whether OSI could be utilized as first-line treatment for NSCLC patients with EGFR-sensitive mutation is worth considering, as no other drugs are currently approved by FDA for OSI-resistant patients. The chemotherapeutics were also therapy options for EGFR TKIs resistant patients [22]. Here, NCI-H1975/OSIR cells were more sensitive to anti-microtubule drug paclitaxel than NCI-H1975 cells, and clinical study also indicated that the OSI-resistant patients showed more sensitive to paclitaxel [30]. Although NCI-H1975/OSIR cells were less resistant anti-metabolite drug pemetrexed, the relatively high concentration of pemetrexed showed weak anti-proliferation in NCI-H1975/OSIR cells. Compared with NCI-H1975 cells, the NCI-H1975/OSIR cells presented more resistance to doxorubicin and fluorouracil, and this resistant effect was not due to the MDR phenotype. Collectively, the paclitaxel could be a therapeutic option for OSI-resistant patients, while the potential mechanism needed to be determined.

Patients resistance to OSI have been observed and several resistant mechanisms of OSI have been identified, such as C797S mutation of EGFR, MET activation, and RAS mutation [23, 31-33]. Unfortunately, no effective drugs or therapy strategies were currently approved for OSI-resistant NSCLC patients. Further identified the resistant mechanisms of OSI in NSCLC patients were necessary for development of new therapeutic drugs or strategies. In the present study, we have identified a novel resistant mechanism of OSI in NCI-H1975/OSIR cells, that is, loss of EGFR expression. Loss of EGFR has been identified as resistant mechanism for gefitinib (a first generation EGFR inhibitor) [10]. This kind of resistant mechanism of OSI suggested that patients might no longer benefit from the EGFR TKIs or EGFR antibody.

The navitoclax is a potent small molecule inhibitor of Bcl-2 family and numerous of studies indicated that navitoclax exhibited therapeutic effect against multiple types of cancers, including lung cancer, acute lymphoblastic leukemia, and ovarian cancer *etc.* [34-36]. So far, various ongoing trials of navitoclax alone or in combination with other drugs were being explored [37-39]. Here, we found that NCI-H197/OSIR cells were more sensitivity to navitoclax and navitoclax-induced cell viability inhibition and apoptosis could be reversed by pretreatment with Z-VAD-FMK. In addition, studies suggested that cells transformed from NSCLC to small cell lung cancer (SCLC) will be sensitivity to navitoclax, and down-regulation of EGFR and RB were biomarkers for this transform [11, 40]. However, the expression of RB protein was similar in both NCI-H1975 and NCI-H1975/OSIR cells (Supplementary Figure S1). More studies should be detected to confirm the transform of NSCLC to SCLC and the therapeutic effect of navitoclax in OSI-resistant cells.

In summary, NCI-H1975/OSIR cells presented lower cell proliferation and higher cell migration and invasion, compared with the parent cells. Cells resistance to OSI showed resistance to other EGFR TKIs gefitinib, erlotinib, afatinib, and rociletinib and chemotherapeutics doxorubicin and fluorouracil, while sensitivity to paclitaxel. The NCI-H1975/OSIR cells did not present MDR phenotype. Loss of EGFR could pose as one of the resistant mechanisms of NCI-H1975/OSIR cells to OSI and navitoclax might be the candidate drug for OSI-resistant NSCLC.

## MATERIALS AND METHODS

### Reagents

OSI, rociletinib, afatinib, erlotinib, gefitinib, pemetrexed, navitoclax, and Z-VAD-FMK were purchased from Selleck Chemicals (Houston, TX, USA). Paclitaxel, doxorubicin, fluorouracil, paraformaldehyde (PFA), MTT, and dimethyl sulfoxide (DMSO) were obtained from Sigma (St. Louis, MO, USA). Crystal violet was purchased from Beyotime Biotechnology Corporation-Shanghai (Shanghai, China). RPMI 1640 medium, fetal bovine serum (FBS), penicillin, streptomycinwere, and phosphate-buffered saline (PBS) were purchased from Gibco Life Technologies (Grand Island, NY, USA). The primary antibodies, *i.e.* PCNA, MMP2, TIMP2, p-FAK (Tyr397), FAK, p-Src (Tyr416), Src, p-Paxillin (Tyr118), Paxillin, P-gp, p-EGFR (Tyr1068), EGFR, p-ERK (Thr202/Tyr204), ERK, p-AKT (Ser473), AKT, c-PARP, c-caspase 3, RB, GAPDH, and the responsive secondary antibodies were obtained from Cell Signaling Technology (Beverly, MA, USA).

### Cell line and culture

NSCLC NCI-H1975 cells (EGFR, L858R and T790M) were obtained from Shanghai Cell Bank (Shanghai, China). Cells were cultured in a RPMI 1640 medium supplemented with 10% (v/v) FBS and antibiotics (100 units/mL penicillin and 100 μg/mL streptomycin). Cells were grown in a 5% CO_2_ incubator at 37 °C.

### Establishment of OSI-resistant NCI-H1975 cells

The OSI-resistant NCI-H1975/OSIR cells were established as described previously [33]. NCI-H1975 cells were exposed to 0.03 μM OSI for 72 h. Cells were then incubated in drug-free medium until the surviving cells had recovered and shown a normal exponential growth rate. The cycles of selection were tested in the presence of gradually increased concentrations of OSI from 0.03 μM to 1.5 μM. After about 6 months, cells became resistant to OSI. Surviving cells were harvested and propagated in drug-free medium. The 21^th^ passage cells were used in the present study. The newly established OSI-resistant cells were indicated as NCI-H1975/OSIR cells. During the establishment of NCI-H1975/OSIR cells, the parental NCI-H1975 cells were always cultured in drug-free medium in parallel.

### MTT assay

The effects of indicated agents on cell viabilities of NCI-H1975 and NCI-H1975/OSIR cells were studied by MTT assay as described in the previous report [41]. Exponentially growing cells were seeded into 96-well plates and cultured to about 70-80%. Cells were treated with indicated concentrations of test compounds. Then, fresh medium with 1 mg/mL MTT was added to the wells and incubated for 4 h at 37 °C. 100 μL of DMSO was added to solubilize the formazan and shook for 10 min in the dark. The absorbance at 570 nm was recorded with a microplate reader (Perkin Elmer, 1420 Multilabel Counter Victor3, Wellesley, MA, USA).

### Colony formation assay

Cells were seeded into 6-well plate at a density of 2,000 per well for NCI-H1975 cells and 3,000 per well for NCI-H1975/OSIR cells. Cells were treated with indicated concentrations of OSI for 72 h and navitoclax for 48 h, respectively. The medium was then replaced with drug-free medium and cells were further incubated for about 7 days. Cells were subsequently fixed using 4% PFA and stained with crystal violet staining solution. Typical images were photographed using an ordinary camera. For quantifying assay, clones were dissolved in acetate acid after crystal violet staining and shook for 10 min in the dark. The absorbance at 590 nm was recorded with a microplate reader (Perkin Elmer, 1420 Multilabel Counter Victor3, Wellesley, MA, USA).

### Morphological change assay

Exponentially growing NCI-H1975 and NCI-H1975/OSIR cells were seeded onto 6-well plate. After incubation at 37 °C for 24 h, the cellular morphology was observed with an AxioCam HRC CCD camera (Carl Zeiss, Germany).

### Western blot assay

Western blot assay was conducted as described previously [42]. Total protein was extracted and the protein concentrations were evaluated with the BCA™ protein assay kit (Pierce, Rockford, IL, USA). Equal quantities of proteins were separated by sodium dodecyl sulfate-polyacrylamide gel electrophoresis, transferred to a PVDF membrane, and blocked with 5% non-fat dry milk. The membrane was then probed with specific primary antibodies overnight at 4 °C. After membranes were washed with PBST, the membranes were incubated with corresponding secondary antibodies at room temperature for 1 h. The specific protein bands were visualized with an ECL advanced western blot analysis detection kit (BD Biosciences, Bedford, MA, USA). Equal protein loading was verified by probing with anti-GAPDH antibodies.

### Migration and invasion assay

The migration and invasion of NCI-H1975 and NCI-H1975/OSIR cells were studied in transwell chambers (10 mm tissue culture transwell with an 8 μm pore size polycarbonate membrane, 24-well companion plate, BD Biosciences, Bedford, MA). The upper chamber of a transwell insert was added with 100 μL PBS for migration assay or 100 μL 1:6 mixture of matrigel (BD Biosciences, Bedford, MA) : PBS for invasion assay and dried for 1 h at 37 °C. The NCI-H1975 or NCI-H1975/OSIR cells were seeded into each well of the upper chamber. The lower chamber was filled 500 μL medium with 10% FBS. After 24 h incubation, non-invading cells that remained on the top aspect of the membrane were removed. The invasive cells attached to the lower surface of the membrane were fixed using 4% PFA at room temperature for 30 min and stained with crystal violet. Random fields were counted and the typical images were photographed by an AxioCam HRC CCD camera (Carl Zeiss, Germany).

### RT-PCR assay

The mRNA expressions of indicated genes were studied with RT-PCR. After incubation of NCI-H1975 and NCI-H1975/OSIR cells in 6-well plate for 24 h, the RNA was extracted with TRIzol reagent (Invitrogen, Carlsbad, CA). The extracted total RNA was reverse-transcribed into single-stranded cDNA using a SuperScript™ III first strand cDNA synthesis kit (Toyobo, Japan). Real-time PCR was performed using SYBR Green PCR Master Mix (Life Technologies) on a Stratagene Mx3005P multiplex quantitative PCR system (Agilent Technologies). The primers of *MRP1*: 5′-*AGAGACAGCTCAGCAGCTCCT*-3′(forward), 5′-*GCC TTGTCAGCCTCCATCAG*-3′(reverse); *MRP7*: 5′-*CCTAG TGCTGACCGTGTTGT*-3′(forward), 5′-*TAGGTTGGCTG CAGTCTGTG*-3′(reverse); *ABCG2*: 5′-*TGATAAATGGAGC ACCGCGA*-3′(forward), 5′-*GCCAGTTGTAGGCTCATCC A*-3′(reverse); *LRP*: 5′-*CCTGTGGATCAGAGGACCC*-3′(forward), 5′-*GCTTCCTGCTCCAGTCTCTG*-3′(reverse) ; *ABCE1*: 5′-*GTTGCCTATCCCTCGTCCAG*-3′(forward), 5′-*TGTCCCCTTTGCAGCCTTAG*-3′(reverse); *ABCF1*: 5′-*GCACTCAAGGGCAAAAAGGG*-3′(forward), 5′-*CAC TTGGCGCTCATACTCCA*-3′(reverse); *EGFR*: 5′-*GAGAG GAGAACTGCCAGAA*-3′(forward), 5′-*GTAGCATTTAT GGAGAGTG*-3′ (reverse); *GAPDH*: 5′-*GCGACACCCAC TCCTCCACCTTT*-3′(forward), 5′-*TGCTGTAGCCAAATTCGTTGTCATA*-3′ (reverse) were prepared by Invitrogen Life Technologies (Shanghai, China).

### Annexin V-FITC and PI staining assay

Apoptotic cells were detected by an annexin V-FITC/PI apoptosis detection kit (BioVision, CA, USA) according to manufacturer's instruction. After incubation with navitoclax with or without pretreatment of Z-VAD-FMK (10 μM, 1 h) for 48 h, NCI-H1975 and NCI-H1975/OSIR cells were trypsinized, washed, and collected. Cells were then suspended in binding buffer with stained by Annexin-FITC and PI solution for 30 min. A total of 10,000 cells were collected and analyzed using a flow cytometer (FACS-Canto, BD Bioscience, USA).

### Statistical analysis

All experiments were repeated at least three times. The mean ± standard deviation was determined for each group. Statistical analysis was performed with one-way analysis of variance of tukey's test or unpaired t test. Differences were considered statistically significant for *P < 0.05 and **P <0.01.

## SUPPLEMENTARY MATERIALS FIGURE


