# Supplementary material for: Characterization of osimertinib (AZD9291)-resistant non-small cell lung cancer NCI-H1975/OSIR cell line
Source: Oncotarget. 2016 Nov 7;7(49):81598–610. doi: 10.18632/oncotarget.13150 (PMC5348415; doi:10.18632/oncotarget.13150)
Supplement: Supplementary file 1 [file oncotarget-07-81598-s001.pdf]

# Characterization of osimertinib (AZD9291)-resistant non-small cell lung cancer NCI-H1975/OSIR cell line

## SUPPLEMENTARY FIGURE

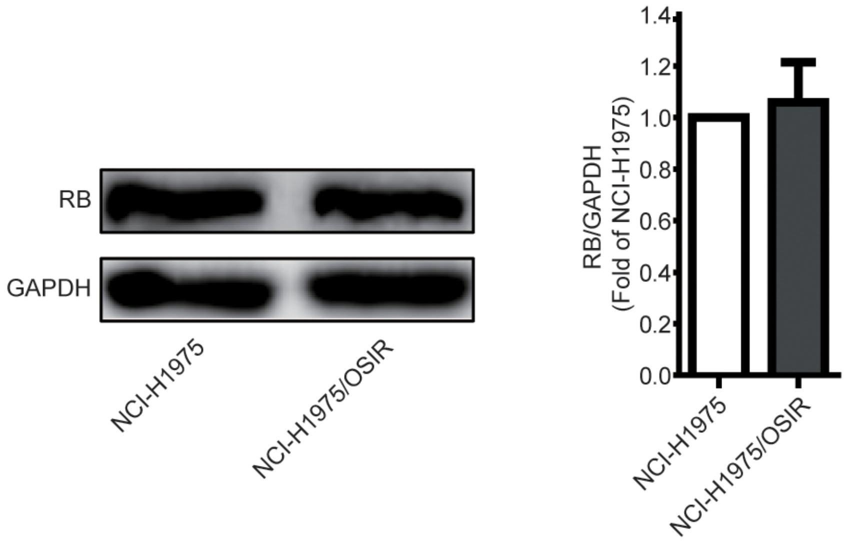


**Supplementary Figure S1: The protein expression of RB in NCI-H1975 and NCI-H1975/OSIR cells was determined by western blot assay.**
